# Tadalafil versus pentoxifylline in the management of diabetic kidney disease: a randomized clinical trial

**DOI:** 10.1186/s13098-024-01363-3

**Published:** 2024-06-24

**Authors:** Sahar Kamal Hegazy, Walaa Ahmed Amaar, Wafaa Salah Mohamed Hegab

**Affiliations:** 1 Clinical Pharmacy Department, Faculty of Pharmacy, Tanta University, Tanta, 31527 Al-Gharbia Egypt; 2 National Institute of Diabetes and Endocrinology of General Organization for Teaching Hospitals and Institutes, Cairo, Egypt

**Keywords:** Diabetic kidney disease, Hemoglobin A1C, Pentoxifylline, Tadalafil, Urinary Albumin/Creatinine ratio

## Abstract

**Aims:**

To investigate the efficacy and safety of tadalafil (TAD) versus pentoxifylline (PTX) in the management of diabetic kidney disease (DKD). Some animal studies and clinical trials reported that tadalafil and pentoxifylline have a reducing effect on different blood glucose parameters and lipid profiles which contribute to progress the patients with diabetes mellitus (DM) to DKD**.**

**Methods:**

From February 2022 to March 2023, 90 patients with type 2 DM and DKD (micro-albuminuria) were enrolled in this randomized-controlled study. The patients were randomized into three equal groups: control group, TAD group, and PTX group. The three groups received traditional blood glucose lowering therapy + ramipril 10 mg PO. The TAD group also received tadalafil 20 mg PO every other day. The PTX group also received pentoxifylline 400 mg PO twice daily.

**Results:**

Both TAD and PTX groups produced statistically significant improvement in the primary outcomes by a significant reduction in Urinary albumin/creatinine ratio (UACR) which was pronounced by a reduction percentage of—47.47%, −53.73% respectively. In addition to a significant decrease in Hemoglobin A1C (HbA_1c_) (mmol/mol), Fasting blood glucose (FBG), 2-h postprandial blood glucose (2-h PPG) (p < 0.001). Only the PTX group showed a significant increase in Cr Cl and a significant decrease in S. Cr (p < 0.001). Only the TAD group showed a significant increase in high-density lipoprotein—cholesterol (HDL-C) (p < 0.001), while the PTX group showed a significant decrease in low-density lipoprotein—cholesterol (LDL-C) (p-value 0.011), and triglyceride (p-value 0.002). Both TAD and PTX groups showed a decrease in tumor necrosis factor-α (TNF-α) which was significant only in the PTX group (p < 0.001). There was a significant increase in malondialdehyde (MDA) (p < 0.001), and an increase in urinary neutrophil gelatinase-associated Lipocalin (uNGAL) (p-value 0.850, 0.014 respectively) which was significant only in the PTX group.

**Conclusions:**

The use of tadalafil or pentoxifylline may serve as an effective adjuvant therapy for patients with diabetic kidney disease.

*Trial Registration*: ClinicalTrials.gov identifier NCT05487755, July 25, 2022.

**Supplementary Information:**

The online version contains supplementary material available at 10.1186/s13098-024-01363-3.

The purpose and hypothesis of this study are to investigate and evaluate the efficacy and safety of TAD versus PTX in the management of DKD.

**What is already known about this subject?**

- Diabetic nephropathy is a serious major microvascular complication of DM and there is a strong association with cardiovascular morbidity and mortality.
- It is the leading cause of end-stage renal disease (ESRD) with poor quality of life that requires renal replacement therapy.

**What is the key question?**

- Do the early diagnosis and early appropriate treatment of the disease can decrease severity and progress to advanced stages?

**What are the new findings?**

- Early diagnosis and early treatment of the disease are critical to prevent disease progression.
- Due to poor knowledge of patients’ diagnosis the disease almost occurs in severe and advanced stages which significantly affects their health.
- Discover new treatments that affect multiple pathways that contribute to the disease progression which have a promising good result on the disease development, progression, and severity.

**How might this impact on clinical practice in the foreseeable future?**

It is so important to use new treatments especially since they have promising good results which may have a good impact on the disease progression and the overall health of the patients rather than the traditional ones which have modest results.

## Introduction

Diabetic kidney disease (DKD) is one of the major microvascular complications of diabetes mellitus DM and the leading cause of end-stage renal disease (ESRD) that requires renal replacement therapies [1]. According to recent estimations, more than 40% of diabetic patients, especially type 2 DM may develop DKD [2].

DKD is clinically defined by persistent elevated urinary albumin to creatinine ratio (UACR) ≥ 30 mg/g and /or decline in kidney function represented by a reduction in estimated glomerular filtration rate (eGFR) < 60 ml/ min /1.73 m^2^ [3]. Urinary albumin creatinine ratio UACR ≥ 30–300 mg/g is micro-albuminuria of DKD or incipient diabetic nephropathy [4]. Pathogenesis of diabetic kidney disease is complex and multi-factorial in which DM has more than a pathway for initiation and progression of the disease [5].

Several risk factors contribute to developing DKD with DM, such as hypertension, obesity, and hyperlipidemia [6]. Although renin–angiotensin–aldosterone system (RAAS) blockade is an established standard care of DKD, albuminuria and progression of renal disease are not completely halted by these agents, and in large studies blockade of RAAS with ACEI or ARBs attenuate the decrease of kidney function by no more than 15–40% [7].

Excess intracellular glucose has been shown to activate cellular signaling pathways such as diacylglycerol (DAG), protein kinase C (PKC) pathway, advanced glycation end-products (AGE), and oxidative stress, these pathways were linked to key steps in the development of glomerulosclerosis [8]. Pro-inflammatory Cytokines (TNF-α, IL-6, IL-1beta, and IL-18) have been linked to the development of DKD [9].

It has been reported that a direct and significant association occurs between serum TNF-α and urinary protein excretion in diabetic patients with normal renal function and micro-albuminuria, as well as patients with renal insufficiency and macro-albuminuria [10]. Oxidative stress and the formation of reactive oxygen species (ROS) lead to activating pathogenic signaling pathways such as the hexamine pathway and polyol pathway which leads to cellular dysfunction, inflammation, apoptosis, and fibrosis [11].

Moreover, ROS has a role in damaging the glomerular filtration barrier which leads to albuminuria [12]. Endothelial dysfunction which is a common feature in DM defined by decreased nitric oxide NO and increased endothelin-1 ET-1, ET-1 is reported to be involved in the pathogenesis of DKD, NO is postulated to play a protective role towards ischemic injuries of renal vascular tissues, action mediated via cyclic guanosine monophosphate- protein kinase G (cGMP-PKG) signaling pathway which initiates renal vascular smooth muscle relaxation [13].

Hyperlipidemia is another risk factor for the development of DKD, in particular, elevated triglycerides TG, elevated low-density lipoprotein cholesterol LDL-C, or decreased high-density lipoprotein cholesterol HDL-C levels, are associated with the development of DKD in both types 1, type 2 DM [14].

TAD is a phosphodiesterase type 5 enzyme (PDE5) inhibitor class of drugs targeting endothelial dysfunction through an increase in nitric oxide-cyclic guanosine monophosphate-protein kinase G (NO-cGMP-PKG) signaling pathway resulting in the relaxation of vascular smooth muscles and vasodilation and increase blood flow, this drug class is currently used in the clinic for the treatment of erectile dysfunction, pulmonary hypertension, and lower urinary tract symptoms [15, 16].

PTX is a non-selective phosphodiesterase inhibitor that mainly inhibits PDE3, and PDE4 resulting in increased cyclic adenosine monophosphate (cAMP), and activation of protein kinase A (PKA) signaling pathway, it is widely used for the treatment of intermittent claudication [17]. Recently, data derived from animal studies and clinical trials about the use of PTX in the management of DKD and the results were promising [18].

Management of DKD by

1. Strict blood glucose control by blood glucose lowering agent oral hypoglycemic agent or insulin or both.
2. Strict blood pressure control.
3. Using RAAS blockade by ACEI to reduce albuminuria and blood pressure.
4. Treatment of hyperlipidemia.
5. Lifestyle adjustments such as weight reduction, and exercise.

Primary outcomes were achieved

1. Adequate blood glucose control.
2. Improve kidney function by decreasing serum creatinine and increasing creatinine clearance.
3. Reduce urinary albumin excretion by reducing UACR which led to normalization of UACR in 40% of patients in the TAD group, and 50% of patients in the PTX group.

Secondary outcomes were achieved

1. Decrease hyperlipidemia by reducing lipid profile LDL, TG (in PTX group only), and increase HDL.
2. Reduce inflammatory marker TNF-α, small increase in oxidative stress marker MDA.

## Methods

### Study design

A randomized, controlled, open-labeled method for (patients, research, and statistical analysis), parallel, prospective clinical trial was conducted from February 2022 to March 2023. Patients were recruited from the outpatient clinic, of the National Institute of Diabetes and Endocrinology, Cairo, Egypt. The study was conducted in collaboration with the Clinical Pharmacy Department, Faculty of Pharmacy, and Tanta University, Tanta, Egypt.

The study protocol was approved by The Research Ethics Committee of The General Organization for Teaching Hospitals and Institutes, Cairo, Egypt, and was carried out in compliance with the Declaration of Helsinki. Before being enrolled in the Trial, all patients gave their informed consent. The trial was registered in Clinical Trials. Gov. under the name "Investigational and Comparative Study to Assess Safety and Effectiveness of Tadalafil and Pentoxifylline in the Management of Diabetic Nephropathy" its identifier is (NCT05487755).

### Inclusion criteria

A confirmed clinical diagnosis of T2DM with a duration of at least 7 years to ensure the establishment of micro-vascular complication DKD, females’ post-menopause, males with sufficient erectile function, patients with DKD with evidence of persistent micro-albuminuria urinary ACR ≥ 30–300 mg/g in 3 consecutive measurements in 6 months period despite treatment with ACEI ramipril 10 mg PO for at least 6 months period before enrollment in the study at maximum recommended tolerated dose. All abnormal results of UACR must be confirmed in two out of three samples at least collected over 6 months before being enrolled in the study.

### Exclusion criteria

Type 1 diabetes mellitus, cardiovascular disease [angina, arrhythmias, myocardial infarction, heart failure (NYHA II–IV), uncontrolled hypertension > (170\100 mm Hg), severe hypotension < (90\50 mm Hg)], hearing problem, vision defect, previous episodes of retinal/ cerebral hemorrhage, psychiatric disease, thyroid disorders, alcohol abuse, smoking, hepatic insufficiency child -Pugh class C, (ALT or AST > 3N), cholestasis, history of (acute or chronic inflammatory, immunologic, malignant, infectious) disease in the previous 3 months, renal disease (acute kidney injury, recent exposure to radio- contrast media, creatinine clearance < 30 ml/ min/1.73 m^2^), bleeding disorders, peptic ulcer, stroke, pregnancy, lactation, known allergy to tadalafil or methylxanthine or taking (other phosphodiesterase enzyme inhibitors PDEI drugs, medications strongly alter CYP3A4 inducer or inhibitor, nitrates, alpha one blockers, immunosuppressive treatment, lipid lowering agent, anti-oxidant drugs) 3 months before enrollment in the study.

**Fig. 1:**
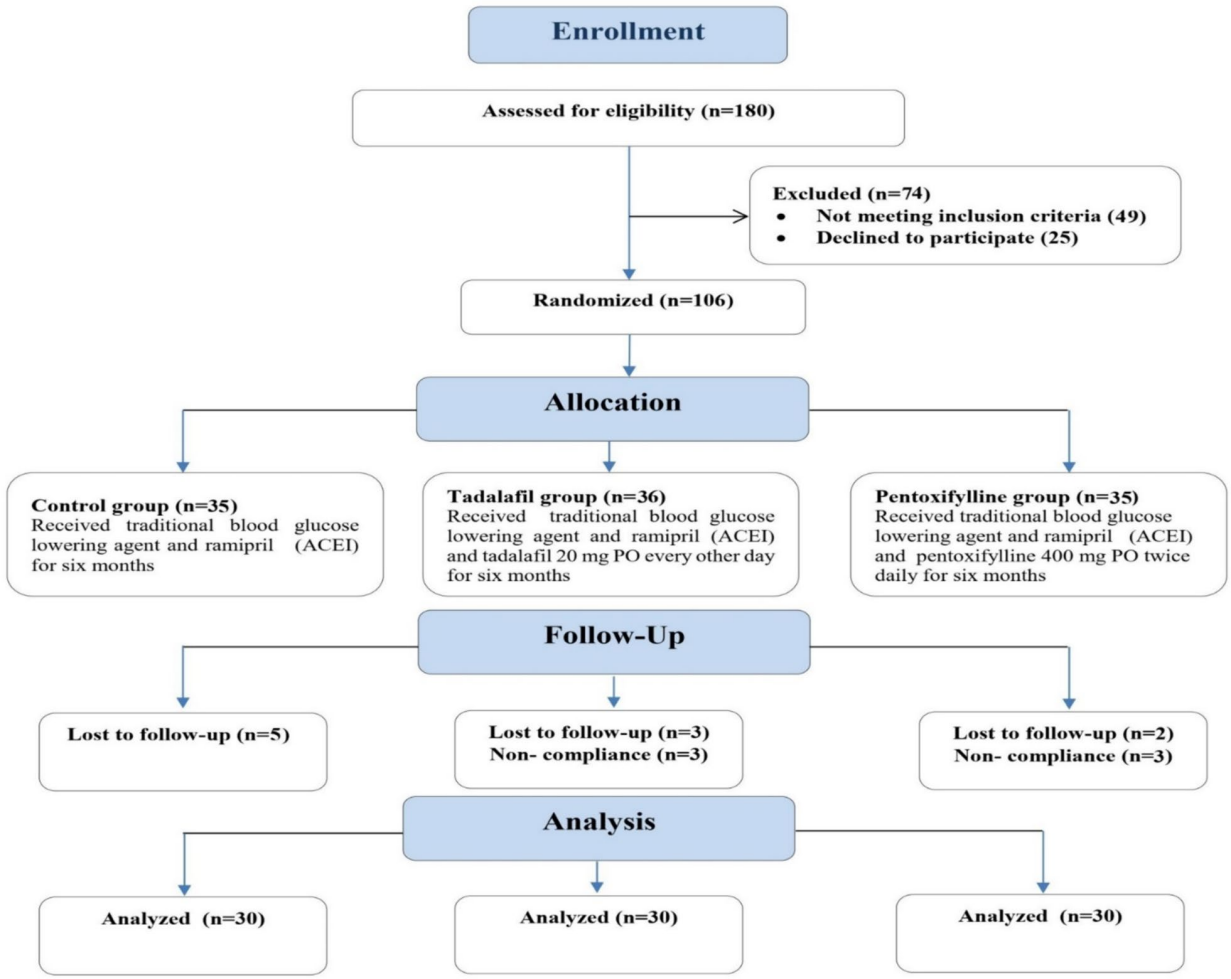
Consort flow diagram

A total number of 90 patients were recruited in the study, randomly divided into three groups with a ratio of 1:1:1 using computer-generated code into three groups. Patients who fulfilled the selection criteria as shown in (Fig. 1) were divided into three equal groups; Group 1 (control group n = 30) received traditional therapy blood glucose lowering agent + ramipril 10 mg PO once daily, Group 2 (TAD group n = 30) received traditional therapy blood glucose lowering agent + ramipril 10 mg PO once daily + tadalafil 20 mg PO every other day, Group 3 (PTX group n = 30) received traditional therapy blood glucose lowering agent + ramipril 10 mg PO once daily + pentoxifylline 400 mg PO twice daily, the study duration was 6 months.

All patients submitted to the following analysis at the baseline and 6 months after the assigned treatment.

Primary outcomes are [FBG, 2-h PPG, HbA1c%, HbA1c (m mol/mol), UACR, S. Cr, and calculated Cr Cl].

Secondary outcomes are [lipid profile (TG, LDL-C, and HDL-C), TNF-α, MDA, and uNGAL].

### Assessment and monitoring

Patients were followed up at monthly intervals for assessment of (compliance, adverse events, and tolerability) to study medications, and monitoring blood pressure. Patients were not allowed to change their (medications, usual diet, or physical activity) during the study period.

### Laboratory methods

At baseline and after 6 months 15 ml of venous blood samples were collected after (10–12) hr. fasting period before breakfast and 2 ml of venous blood samples were collected after 2 h from the start of a meal for measure 2-h PPG. Two ml of the 15 ml of blood samples were transferred into EDTA test tubes for immediate measurement of HbA1c% using the chromatography method (HPLC: ion exchange chromatography). The remaining blood was centrifuged for 15 min at 3000 rpm. The separated serum was divided into two portions. The first portion was used for the immediate measurement of FBG, S. Cr, and lipid profile using the colorimetric method, while the second portion was frozen at −80 °Cfor measuring serum (TNF-α) and serum malondialdehyde (MDA) using enzyme-linked immunosorbent assay (ELISA) kits. Creatinine clearance was calculated using Cockcroft-Gault Formula: creatinine clearance (ml/min) = [(140—age (years)] × weight (kg)/[72 × serum creatinine (mg/dl)] × (0.85 if female) [19].

The postprandial blood samples which were collected after 2 h from the start of a meal were centrifuged for 15 min at 3000 rpm then the separated serum was taken for immediate measurement of (2-h PPG) using the enzymatic colorimetric method. Urine samples were collected in sterile containers, first void morning urine samples, and then centrifuged for 10 min at 3000 rpm the clear supernatant was divided into three portions; the first was used for measurement of urinary albumin using the turbidimetric immunoassay method. The second portion was for measurement of urinary creatinine after (dilute 1 ml urine + 49 ml with distilled water) using the colorimetric method and then calculating ratio (urine albumin value to urine creatinine value) (UACR), and the third portion was frozen at − 80 °C for measurement of urinary NGAL using enzyme-linked immunosorbent assay (ELISA) kits.

### Statistical analysis

Data analysis was performed using SPSS statistical package version 24.0 (March 2016), IBM corporation software group, USA. Qualitative data were described as number and percent. Normally distributed data (quantitative) were described as mean ± standard deviation (SD). The Kolmogorov–Smirnov test was used to check the normality of the distribution of quantitative data. The chi-square test was applied to compare qualitative categorical clinical variables between groups. Significant variation and the Percentage change in variables within each group between baseline and 6 months after treatment were detected using the paired Student t-test. Significant variation in variables among all groups at baseline and 6 months after treatment was detected using the one-way ANOVA test followed by post hoc (Tukey's) test. Pearson’s correlation test assessed the correlation among the measured parameters. P < 0.05 were considered statistically significant.

## Results

The whole sample was randomized, controlled, open-labeled, parallel, all demographic and clinical data of the whole sample was shown in Table 1.

**Table 1:** Demographic and clinical data of the studied patients

Variable		Control group (n = 30)	Tadalafil group (n = 30)	Pentoxifylline group (n = 30)	P value
Age (Years)		57.73 ± 5.77	54.83 ± 7.07	56.96 ± 6.66	0.210
Gender	Male	6 (20%)	10 (33.3%)	9 (30%)	0.487
	Female	24 (80%)	20 (66.67%)	21 (70%)	
BMI (kg/m^2^)	Normal (18.5–24.9)	3 (10%)	2 (6.67%)	3 (10%)	0.945
	Overweight (25–29.9)	25 (83.33%)	27 (90%)	25 (83.33%)	
	Obese ≥ 30	2 (6.67%)	1 (3.33%)	2 (6.67%)	
Duration of DM (Years)		9.03 ± 1.88	8.93 ± 1.96	8.93 ± 1.96	0.974
Treatment of DM	• Oral hypoglycemic agent	7 (23.33%)	9 (30%)	3 (10%)	0.306
	• Insulin	13 (43.33%)	12 (40%)	12 (40%)	
	• Oral hypoglycemic agent and insulin	10 (33.33%)	9 (30%)	15 (50%)	
Hypertensive		19 (63.33%)	13 (43.33%)	18 (60%)	0.248
Normotensive		11 (36.67%)	17 (56.67%)	12 (40%)	
HTN duration (Years)		2.89 ± 0.99	3.15 ± 0.98	3.16 ± 0.92	0.642
Systolic blood pressure (mm Hg)		128 ± 9.15	125.66 ± 9.07	128.5 ± 10.01	0.465
Diastolic blood pressure (mm Hg)		79.66 ± 6.94	78.16 ± 7.13	80.66 ± 7.16	0.391

Data was expressed as number (%) or mean ± SD

*DM* Diabetes mellitus, *HTN* Hypertension

^*^p < 0.05 was considered statistically significant, the statistical tests used in the table (ANOVA test, chi-square test)

### Demographic and clinical data of the studied patients

There was no significant difference in demographic or anthropometric parameters between the studied groups at baseline. (Table 1). Table 2 shows the effect of the intervention on the biochemical parameters of the primary outcomes, at baseline no significant difference was detected among the three studied groups in all measured parameters, after 6 months.

**Table 2:** The results of the biochemical parameters of the primary outcomes

	Control group (N = 30)	Tadalafil group (N = 30)	Pentoxifylline group (N = 30)	P value
UACR (mg/g) Baseline	109.93 ± 47.82	109.87 ± 65.49	120.41 ± 79.16	0.773
After 6 Months	163.41 ± 64.33	43.79 ± 25.61^#^	46.19 ± 33.43^#^	< 0.001*
P-value within each group	< 0.001*	< 0.001*	< 0.001*	–
HbA1c (m mol/mol) Baseline	87.700 ± 19.834	89.967 ± 19.425	99.900 ± 22.536	0.418
After 6 Months	103.200 ± 25.397	72.633 ± 20.819^#^	72.300 ± 15.594^#^	< 0.001*
P-value within each group	< 0.001*	< 0.001*	< 0.001*	–
HbA1c% Baseline	10.16 ± 1.81	10.36 ± 1.78	10.74 ± 1.46	0.418
After 6 Months	11.59 ± 2.32	8.78 ± 1.90^#^	8.78 ± 1.42^#^	< 0.001*
P-value within each group	< 0.001*	< 0.001*	< 0.001*	–
FBG (mg/dL) Baseline	215.06 ± 77.73	220.43 ± 63.96	232.43 ± 72.72	0.632
After 6 Months	238.50 ± 97.13	171.65 ± 67.30^#^	170.47 ± 54.17^#^	0.001*
P-value within each group	0.010*	< 0.001*	< 0.001*	–
2-h PPG (mg/dL) Baseline	322.30 ± 100.41	317.30 ± 79.18	350.26 ± 104.49	0.357
After 6 Months	367.36 ± 100.41	245.63 ± 98.23^#^	223.60 ± 63.83^#^	< 0.001*
P-value within each group	< 0.001*	< 0.001*	< 0.001*	–
Cr Cl (ml/min/1.73m^2^) Baseline	77.12 ± 21.11	83.10 ± 18.24	76.72 ± 16.41	0.339
After 6 Months	59.79 ± 13.12	89.52 ± 21.92^#^	92.32 ± 19.99^#^	< 0.001*
P-value within each group	< 0.001*	0.126	< 0.001*	–
S.Cr (mg/dL) Baseline	0.95 ± 0.32	0.93 ± 0.27	0.97 ± 0.24	0.832
6 months after	1.18 ± 0.31	0.86 ± 0.23^#^	0.80 ± 0.20^#^	< 0.001*
P-value within each group	< 0.001*	0.134	< 0.001*	–

Data was expressed as mean ± SD, the statistical tests used in the table (paired student t-test, ANOVA test)

*Cr Cl* Creatinine clearance, *FBG* Fasting blood glucose, *HbA1c%* Hemoglobin A1C %, HbA1c (mmol/mol), *2-h* *PPG* 2-h postprandial blood glucose, *S.Cr* Serum creatinine, *UACR* Urinary albumin to creatinine ratio

^*^p < 0.05 was considered statistically significant

^#^significant compared to control group(ANOVA/post Hoc test)(p < 0.05)

### Effect on UACR

**Fig. 2:**
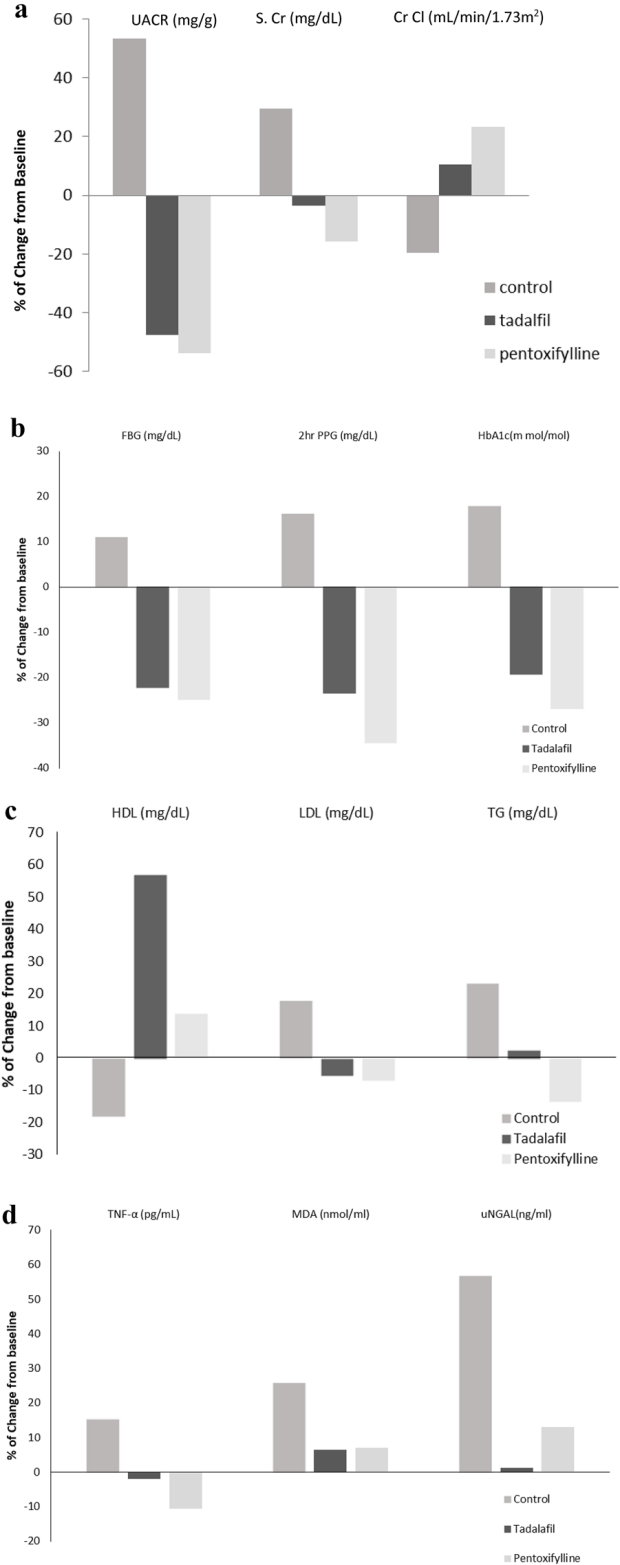
**a** Percent change in UACR, S.Cr, Cr Cl from baseline to 6 months in all study groups. *UACR* Urinary albumin/creatinine ratio, *S.Cr* Serum creatinine, *Cr Cl* Creatinine clearance. **b** Percent change in FBG, 2-h PPG, HbA1c(m mol/mol) from baseline to 6 months in all study groups. *FBG* Fasting blood glucose, *2-h PPG* 2-h postprandial blood glucose, *HbA1c* Hemoglobin A1C. **c** Percent change in HDL, LDL, TG from baseline to 6 months in all study groups. *HDL* High-density lipoprotein, *LDL* Low-density lipoprotein, *TG* Triglycerides. **d** Percent change in TNF- α, MDA, uNGAL from baseline to 6 months in all study groups. *TNF- α* Tumor necrosis factor-α, *MDA* Malondialdehyde, *uNGAL* Urinary neutrophil gelatinase-associated Lipocalin

Both TAD and PTX groups showed a significant reduction in UACR compared to their baseline value by -47.47%, and -53.73% respectively, while the control group showed a significant increase in UACR by 53.13% compared to its baseline value. (Table 2) (Fig. 2a).

- There was a significant difference between the control group and both TAD and PTX groups regarding the UACR level and the effect of both TAD and PTX was comparable in reduction of UACR level.

### Effect on blood glucose

Both TAD and PTX groups showed a significant reduction in [HbA1c (mmol/mol), FBG, 2-h PPG] compared to their baseline values. For the TAD group -19.30%, -22.29%, -23.43% respectively. And for the PTX group -26.92%, -24.82%, -34.47% respectively while the control group showed a significant increase in [HbA1c (m mol/mol), FBG, 2-h PPG] compared to its baseline value by 17.77%, 10.96%, 16.13% respectively. (Table 2), (Fig. 2b).

- There was a significant difference between the control group and both TAD and PTX groups regarding [HbA1c (m mol/mol), FBG,2-h PPG] and the effect of both TAD and PTX groups was comparable in reduction of [HbA1c (m mol/mol), FBG, 2-h PPG] levels.

### Effect on kidney function

Both TAD and PTX groups showed an increase in Cr Cl compared to their baseline values by 10.49%, and 23.18% respectively which was significant only in the PTX group, while the control group showed a significant reduction in Cr Cl compared to its baseline value by -19.59%. (Table 2), (Fig. 2a).

Both TAD and PTX groups showed a decrease in S. Cr compared to their baseline values by -3.63%, -15.69% respectively, which was significant only in the PTX group, while the control group showed a significant increase in S. Cr compared to its baseline value by 29.29%. (Table 2), (Fig. 2a).

Table 3 shows the effect of the intervention on the biochemical parameters of the secondary outcomes. At baseline no significant difference was detected among the three studied groups in all measured parameters, after 6 months.

**Table 3:** The results of the biochemical parameters of the secondary outcomes

	Control group (N = 30)	Tadalafil group (N = 30)	Pentoxifylline group (N = 30)	P value
HDL-C (mg/dL) Baseline	36.93 ± 9.18	40.68 ± 18.37	43.25 ± 15.91	0.265
After 6 months	29.46 ± 6.30	56.00 ± 16.34^#**$**^	46.03 ± 18.25^#^	< 0.001*
P-value within each group	< 0.001*	< 0.001*	0.451	–
LDL- C(mg/dL) Baseline	173.97 ± 18.35	179.42 ± 51.88	165.40 ± 44.60	0.412
After 6 Months	204.43 ± 26.95	164.88 ± 46.90^#^	150.41 ± 40.15^#^	< 0.001*
P-value within each group	< 0.001*	0.095	0.011*	–
TG (mg/dL) Baseline	218.33 ± 37.15	230.61 ± 97.48	212.98 ± 83.14	0.663
After 6 Months	268.68 ± 52.07	215.03 ± 85.08^#^	178.22 ± 66.13^#^	< 0.001*
P-value within each group	< 0.001*	0.291	0.002*	–
TNF-α (pg/mL) Baseline	137.62 ± 20.00	139.73 ± 18.06	141.29 ± 17.14	0.743
After 6 Months	158.78 ± 25.74	137.08 ± 20.18^#^	126.30 ± 16.76^#^	< 0.001*
P-value within each group	< 0.001*	0.165	< 0.001*	–
MDA (nmol/ml) Baseline	17.34 ± 2.79	17.09 ± 3.56	16.43 ± 2.23	0.463
After 6 Months	21.73 ± 3.60	18.16 ± 3.80^#^	17.55 ± 2.22^#^	< 0.001*
P-value within each group	< 0.001*	< 0.001*	< 0.001*	
uNGAL (ng/ml) Baseline	397.20 ± 87.66	405.38 ± 78.12	399.79 ± 120.31	0.946
After 6 Months	600.49 ± 124	399.61 ± 160.08^#^	443.81 ± 132.25^#^	< 0.001*
P-value within each group	< 0.001*	0.850	0.014*	–

Data was expressed as mean ± SD, the statistical tests used in the table (paired student t-test, ANOVA test)

*HDL-C* High-density lipoprotein–cholesterol, *LDL-C* Low-density lipoprotein-cholestroL, *MDA* Malondialdehyde, *TG* Triglycerides, *TNF-α* Tumor necrosis factor-α, *uNGAL* Urinary neutrophil gelatinase-associated Lipocalin

^*^p < 0.05 was considered statistically significant

^#^significant compared to control group (ANOVA/post Hoc test) (p < 0.05)

^**$**^significant compared to other intervention groups (ANOVA/post Hoc test) (p < 0.05)

### Effect on lipid profile

Both TAD and PTX groups showed an increase in HDL-C compared to their baseline value by 56.69%, and 13.61% respectively which was significant only in TAD group, while the control group showed a significant reduction in HDL-C compared to its baseline value by −18.04%, both TAD and PTX groups showed a decrease in LDL-C compared to their baseline value by −5.26%, −6.75% respectively which was significant only in PTX group, while the control group showed a significant increase in LDL-C compared to its baseline value by 17.74%, PTX group showed a significant reduction in TG compared to its baseline value by −13.40%, both TAD and control groups showed an increase in TG compared to their baseline value by 2.57%, 23.13% respectively which was significant only in the control group. (Table 3), (Fig. 2c).

- There was a significant difference between the control group and both TAD and PTX groups regarding HDL-C, LDL-C, and TG.

Effect on inflammatory biomarker TNF-α and oxidative stress biomarker MDA. Regarding TNF-α both TAD and PTX groups showed a decrease in TNF-α compared to their baseline value by −1.88%, −10.60% respectively which was significant only in the PTX group, while the control group showed a significant increase in TNF-α compared to its baseline value by 15.30%. (Table 3), (Fig. 2d).

Regarding MDA, the TAD, PTX, and control groups showed a significant increase in MDA compared to their baseline value by 6.54%, 7.01%, and 25.92% respectively. (Table 3), (Fig. 2d).

- There was a significant difference between the control group and both TAD and PTX groups regarding TNF-α and MDA.

### Effect on uNGAL biomarker

TAD, PTX, and control groups showed an increase in uNGAL compared to their baseline value by 1.28%, 13.14%, and 56.68% respectively which was significant in PTX and control groups. (Table 3), (Fig. 2d).

- There was a significant difference between the control group and both TAD and PTX groups regarding uNGAL.

**Fig. 3:**
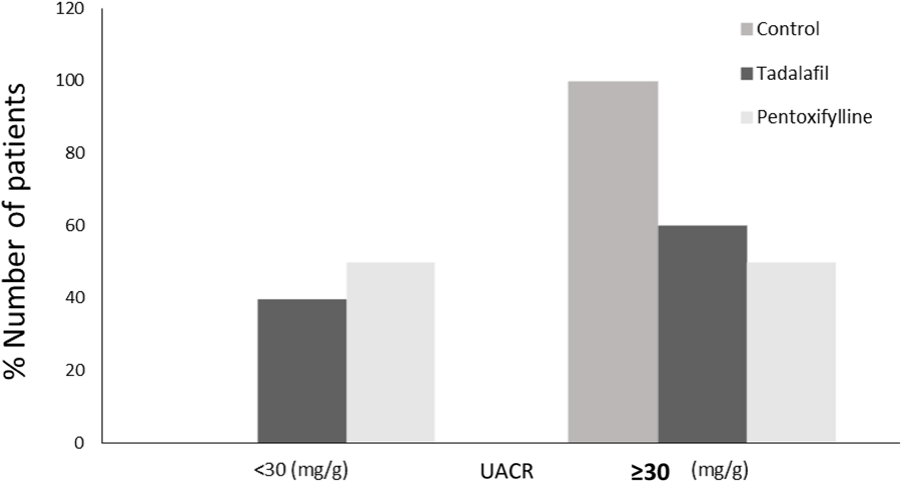
Effect of (Tadalafil, Pentoxifylline) on normalization of micro-albuminuria

Effect of the intervention on normalization of micro-albuminuria. Normalization of micro-albuminuria is one of the primary outcomes of this research. Table 4, Fig. 3 shows that (50% of patients in the PTX group and 40% of patients in TAD group) achieved this target.

**Table 4:** Effect of (Tadalafil, and pentoxifylline) on normalization of micro-albuminuria

UACR After 6 Months	Control group N = 30	Tadalafil group N = 30	Pentoxifylline group N = 30	P-value
< 30 (mg/g)	0%	12 (40%)	15 (50%)	< 0.001*
≥ 30–300 (mg/g)	30 (100%)	18 (60%)	15 (50%)	

*UACR* Urinary albumin to creatinine ratio. UACR values are divided into two groups either group: UACR < 30 (mg/g) is normal albuminuria, or group: UACR ≥ 30–300 (mg/g) is micro-albuminuria

Data was expressed as (%) number of patients of (control, tadalafil, pentoxifylline) groups included in each group either (UACR < 30(mg/g) or UACR ≥ 30–300 (mg/g)

^*^p < 0.05 was considered statistically significant, p-value between the three studied groups was < 0.001*, the statistical test used in the table (chi-square test)

### Correlation analysis between variables

**Fig. 4:**
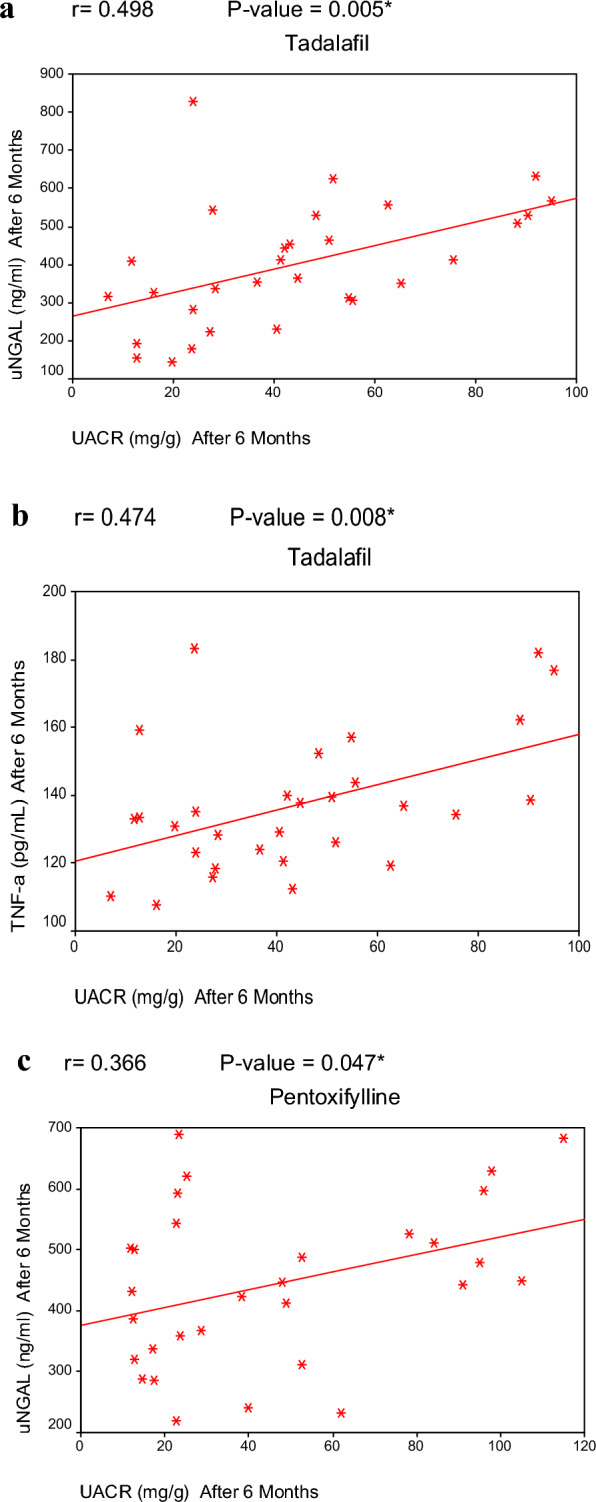
**a** Significant positive correlation between UACR & uNGAL in the Tadalafil group. **b** Significant positive correlation between UACR&TNF-α in the Tadalafil group. **c** Significant positive correlation between UACR & uNGAL in Pentoxifylline group

Pearson’s correlation after the intervention period of 6 months revealed a significant, positive correlation between UACR and uNGAL in both the TAD group and PTX group respectively. (Fig. 4a, c). Moreover, a significant, positive correlation between UACR& TNF-α in TAD group. (Fig. 4b). Reported side effects among all study groups were all included in (Table 5).

**Table 5:** Reported side effects among all study groups

Side effect	Control group N = 30	Tadalafil group N = 30	Pentoxifylline group N = 30	P value
Headache	4 (13.33%)	5 (16.67%)	1 (3.33%)	0.232
Hypotension	3 (10%)	2 (6.67%)	0%	0.227
Dizziness	0%	1 (3.33%)	1 (3.33%)	0.600
Myalgia	0%	3 (10%)	0%	0.045*
Diarrhea	2 (6.67%)	0%	1 (3.33%)	0.355
Abdominal discomfort	0%	0%	4 (13.33%)	0.015*
Bloating	0%	0%	4 (13.33%)	0.015*
Nausea	0%	0%	3 (10%)	0.045*
Vomiting	1 (3.33%)	0%	3 (10%)	0.160

Data was expressed as number (%)

^*^p < 0.05 was considered statistically significant, the statistical test used in the table (chi-square test)

### Side effect analysis

There were no significant differences between the three studied groups, regarding headache, hypotension, dizziness, diarrhea, and vomiting except for myalgia which was prevalent in TAD group and patients were advised to take analgesics on demand not regularly, while abdominal discomfort, bloating, and nausea which were prevalent in PTX group and patients were advised to take medication after meals and not on empty stomach to enhance its tolerability, the two drugs TAD and PTX are well tolerated with self-limited side effects that are temporary and relieved easily (Table 5).

## Discussion

It is the first clinical study to compare the effect of Tadalafil and pentoxifylline in the management of diabetic kidney disease. Proteinuria is a well-known risk factor for cardiovascular mortality and morbidity and is strongly associated with the progression of kidney disease [20]. The degree of albuminuria reduction is linearly related to the subsequent renal protection [21]. The risk for major renal involvements in patients with diabetic kidney disease (micro-albuminuria) is two times greater than in normo-albuminuric patients; this reinforces the necessity of early detection and treatment of micro-albuminuria [22]. Although micro-albuminuria is considered a risk factor for the development of macro-albuminuria, fortunately not all patients progress to macro-albuminuria and some patients may regress to normal albuminuria [23].

Animal studies as well as many clinical trials reported that TAD has different beneficial effects in multiple diseases regarding hyperglycemia [24], inflammation [25], oxidative stress [26] and endothelial dysfunction [27] that make it a target in treatment or at least as adjuvant therapy for multiple diseases. PTX has recently reported various beneficial effects on the kidneys directly [28]. Or through its effect on hyperglycemia [29], inflammation [30] and oxidative stress [31]. Our results revealed that both TAD, and PTX drugs significantly decreased UACR as compared to their baseline values and to control group.

This may be explained by their effect in reducing blood glucose [FBG, 2-h PPG, HbA_1c_ (m mol/mol)], reducing inflammation, improving lipid profile (increase HDL-C, decrease LDL-C) and reducing TG in the PTX group only. This result is consistent with previous studies that reported the effect of TAD and PTX in reducing albuminuria [32].

In the current study, the reduction of UACR in both groups TAD and PTX led to the normalization of UACR by percentage 40%, and 50% in TAD, PTX groups respectively which gives attention to the role of both drugs in the protection and management of DKD. Our results revealed that both TAD, and PTX significantly decreased [HbA_1c_ (m mol/mol), FBG, 2-h PPG] as compared to their baseline values and control group. In agreement with this finding, TAD was reported to reduce both FBG and HbA1C% [33], as well as PTX did [34]. The effect of both drugs in improving kidney function was evident in the current study as indicated by the increase in Cr Cl & the decrease in S. Cr which is in line with other studies [35–37].

Our results revealed that both TAD and PTX have favorable effects on lipid profile which may contribute to DM in the progression of DKD. There was an increase in serum level of HDL-C in TAD, and PTX which was significant only in TAD group compared to its baseline values, to control group, and even to PTX group. TAD was found to be insignificant [decreased LDL-C and increased TG]. Some previous studies demonstrated that TAD has a favorable effect on the lipid profile [38], while others demonstrated that TAD does not affect the lipid profile [39].

Regarding the PTX effect on lipid profile, there was a significant decrease in (LDL-C, and TG) as compared to their baseline values and to control group, and an insignificant increase in HDL-C as compared to its baseline values and TAD group. This result is in accordance with Elseweidy et al. who demonstrated that PTX has a significant effect on all lipid profile parameters TC, TG, HDL-C and LDL-C [40]. TNF-α is a pro-inflammatory cytokine that is cytotoxic to the glomerular and may induce significant renal damage, our result revealed a decrease in serum level of TNF-α significantly in PTX group and non-significantly in TAD group compared to their baseline values, to control group, which is consistent with previous studies [41, 42].

MDA is the end product of lipid peroxidation and a well-known marker for free radical formation and it gives the extent to the degree of damage in tissue [43]. Our result revealed a significant increase in serum level of MDA in both TAD, and PTX groups compared to their baseline values which is inconsistent with previous studies demonstrating that both TAD and PTX have reduced serum levels of MDA [44, 45]. However, the increase in serum level of MDA in both TAD, and PTX groups was still lower than the increase in the control group which may be explained by that both drugs have a protective effect against the highly elevated level of serum MDA in the control group by their beneficial effect on oxidative stress. uNGAL is a marker of structural damage of renal tubules, its level can quantify the degree of tubular damage, it is temporally increased before the appearance of micro-albuminuria and proteinuria [46].

Our result revealed an increase in uNGAL in PTX, TAD, and control groups compared to their baseline values which were significant only in PTX, and control groups, our result is consistent in PTX group and inconsistent in TAD group with the previous studies [47, 48]. Although both TAD, and PTX groups had little increase in uNGAL level it was still lower than the highly elevated level in the control group which can be explained by that both drugs TAD, and PTX have a protective effect against kidney damage reflected by a lesser increase in uNGAL than in the control group, taken in consideration that TAD had superior to PTX in the effect on uNGAL.

However small sample size of the study can't generalize the results of the study to all patients with DM whether they have the same characteristics as patients of the study or are different from them, so further large-scale studies are still required. In the future can the research find new treatments for DM with multiple beneficial effects that improve the overall health of patients and decrease the incidence and the severity of diabetic micro and macro-vascular complications?

## Conclusion

The data obtained from the current study revealed that both tadalafil and pentoxifylline may represent a promising adjuvant therapy in the management of DKD, especially since both drugs showed a significant reduction in blood glucose [FBG, 2-h PPG, HbA_1c_ (m mol/mol)] beneficial effect on inflammation, oxidative stress, and hyperlipidemia, which make them target therapy in DKD and other various diseases by their beneficial effects on more than pathways implicated in different diseases. However, further large-scale, and more longitudinal studies are still required to confirm our results.

### Supplementary Information

Supplementary material 1.

## Data Availability

Data is available upon reasonable request from the corresponding author.

## Abbreviations

DKD: Diabetic kidney disease
Cr Cl: Creatinine clearance
MDA: Malondialdehyde
NO-cGMP-PKG: Nitric oxide- cyclic guanosine monophosphate-protein kinase G
PDE3: Phosphodiesterase enzyme 3
PKA: Protein kinase A
PKC: Protein kinase C
PTX: Pentoxifylline
RAAS: Renin–angiotensin–aldosterone system
ROS: Reactive oxygen spices
TAD: Tadalafil
UACR: Urinary albumin to creatinine ratio
uNGAL: Urinary neutrophil gelatinase associated with Lipocalin
