# Supplementary material for: Tadalafil versus pentoxifylline in the management of diabetic kidney disease: a randomized clinical trial
Source: Diabetol Metab Syndr. 2024 Jun 24;16:138. doi: 10.1186/s13098-024-01363-3 (PMC11194930; doi:10.1186/s13098-024-01363-3)
Supplement: Supplementary file 1 — Supplementary material 1. [file 13098_2024_1363_MOESM1_ESM.docx]

Treatment of T2DM for Patients of the study was divided into three groups.

- Oral hypoglycemic agents
- Insulin
- Oral hypoglycemic agent and insulin

1.oral hypoglycemic agents

| Oral hypoglycemic agent | | Dose | Frequencies |
| --- | --- | --- | --- |
| 1.sulfonylurea  Gliclazide | | 60 mg MR  Up to 120 mg taken orally in a single daily intake | Once daily |
| Dipeptidyl peptidase –Ⅳ (DPP Ⅳ) inhibitors | 1.sitagliptin | 100 mg | Once daily |
|  | 2.linagliptin | 5 mg | Once daily |
| Fixed dose Combination therapy of  sodium –glucose cotransporter-2 (SGLT2) inhibitors  +  Dipeptidyl peptidase –Ⅳ (DPP Ⅳ) inhibitors  (Empagliflozin+Linagliptin) | | 25/5 mg | Once daily |

2.insulin therapy

| Insulin | frequency |
| --- | --- |
| Intermediate -acting human insulin (NPH)  +  short acting human insulin | Once or twice daily  +  Within 30 minutes before meals |
| Pre-mixed biphasic human insulin  or  Pre-mixed biphasic analogue | twice daily within (30 minutes) before eating  or  Immediately before meals |

3. Oral hypoglycemic agent and insulin

Oral hypoglycemic agent

| Oral hypoglycemic agent | | Frequency |
| --- | --- | --- |
| Sulfonylurea | Gliclazide (60mg) MR up to (120 mg) | Once daily |
| Dipeptidyl peptidase –Ⅳ (DPP Ⅳ) inhibitors | 1. sitagliptin (100 mg ,50 mg) | Once daily |
|  | 2. linagliptin 5mg | Once daily |
| sodium –glucose cotransporter-2 (SGLT2) inhibitors | 1. Empagliflozin 25 mg | Once daily |
|  | 2. dapagliflozin (5mg, 10 mg) | Once daily |

Insulin

| Type of insulin | Frequency |
| --- | --- |
| Intermediate - acting human insulin | Once or twice daily |
| Pre-mixed biphasic human insulin  or  Pre-mixed biphasic analogues | Twice daily  or  Immediately before meals |

**Notes**

1. Dosage of oral hypoglycemic agent was individualized according to each patient (HbA1C%, blood glucose levels, kidney function (creatinine clearance CrCl ml/ min / 1.73 m^2^, degree of hepatic impairment if any present).
2. Side effects of oral hypoglycemic agents for example (hypoglycemia,weight gain …. etc) were monitored during the study and appropriate action if any present was taken.
3. Dosage of insulin (units)for each patient was individualized according to each patient (weight, HbA1C%, blood glucose level, kidney function (creatinine clearance CrCl ml/ min / 1.73 m^2^, diet, life style).
4. Side effects of insulin for example (hypoglycemia)were monitored during study and appropriate action was taken if any present.
